# Rethinking pioglitazone as a cardioprotective agent: a new perspective on an overlooked drug

**DOI:** 10.1186/s12933-021-01294-7

**Published:** 2021-05-18

**Authors:** Lorenzo Nesti, Domenico Tricò, Alessandro Mengozzi, Andrea Natali

**Affiliations:** 1 Metabolism, Nutrition, and Atherosclerosis Laboratory, Department of Clinical and Experimental Medicine, University of Pisa, Via Savi 10, 56126 Pisa, Italy; 2 Cardiopulmonary Laboratory, Department of Clinical and Experimental Medicine, University of Pisa, Pisa, Italy; 3 Department of Surgical, Medical and Molecular Pathology and Critical Care Medicine, Pisa, Italy; 4 Institute of Life Sciences, Sant’Anna School of Advanced Studies, Pisa, Italy

**Keywords:** Type 2 diabetes, Cardiovascular, Pharmacologic effects, PPARs, Pioglitazone, Cardiovascular risk factors, Cardiovascular prevention, Clinical management, Cardioprotection, Heart failure

## Abstract

Since 1985, the thiazolidinedione pioglitazone has been widely used as an insulin sensitizer drug for type 2 diabetes mellitus (T2DM). Although fluid retention was early recognized as a safety concern, data from clinical trials have not provided conclusive evidence for a benefit or a harm on cardiac function, leaving the question unanswered. We reviewed the available evidence encompassing both in vitro and in vivo studies in tissues, isolated organs, animals and humans, including the evidence generated by major clinical trials. Despite the increased risk of hospitalization for heart failure due to fluid retention, pioglitazone is consistently associated with reduced risk of myocardial infarction and ischemic stroke both in primary and secondary prevention, without any proven direct harm on the myocardium. Moreover, it reduces atherosclerosis progression, in-stent restenosis after coronary stent implantation, progression rate from persistent to permanent atrial fibrillation, and reablation rate in diabetic patients with paroxysmal atrial fibrillation after catheter ablation. In fact, human and animal studies consistently report direct beneficial effects on cardiomyocytes electrophysiology, energetic metabolism, ischemia–reperfusion injury, cardiac remodeling, neurohormonal activation, pulmonary circulation and biventricular systo-diastolic functions. The mechanisms involved may rely either on anti-remodeling properties (endothelium protective, inflammation-modulating, anti-proliferative and anti-fibrotic properties) and/or on metabolic (adipose tissue metabolism, increased HDL cholesterol) and neurohormonal (renin–angiotensin–aldosterone system, sympathetic nervous system, and adiponectin) modulation of the cardiovascular system. With appropriate prescription and titration, pioglitazone remains a useful tool in the arsenal of the clinical diabetologist.

## Introduction

Early after their approval for the treatment of type 2 diabetes (T2D) in 1985, the insulin-sensitizing agents thiazolidinediones (TDZs) *pioglitazone*, *rosiglitazone* and *troglitazone* displayed alleged safety concerns about fluid retention, increased risk of developing heart failure (HF), ischemic heart disease, and liver toxicity -not all substantiated by later observations-, determining troglitazone and rosiglitazone withdrawal and subsequent reinstatements [1]. Moreover, concerns related to potential adverse effects of pioglitazone, including weight gain, bladder cancer and decreased bone mineral density with increased risk of fractures, have led to a progressive and sustained decline in pioglitazone prescriptions [2–4]. Nevertheless, treatment with pioglitazone is still available in most countries, is cost-effective, and has gained renewed popularity after novel favorable evidence [5, 6]. Especially after the discovery of the cardioprotective effects of sodium-glucose cotransporter (SGLT-2) inhibitors and glucagon-like peptide (GLP)-1 receptor agonists [7], the T2D treatment paradigm has faced a Copernican revolution, now also focusing on non-glycemic, cardioprotective effects of glucose-lowering agents -including the oldest ones [8]. Despite the initial aversion due to increased HF risk, several studies have examined the complex metabolic and biological roles of pioglitazone in many cardiovascular diseases. Experimental and human data suggest beneficial effects on the vascular system, including delayed atherosclerosis progression and reduced cardiovascular events [9]. Although pioglitazone might worsen HF by inducing sodium-water retention and oedema, there are some counterarguments [10] : first, it does not increase mortality due to HF, second, it has no detectable deleterious effect on the heart [11, 12]. On the contrary, it positively modulates several cardiac and vascular functions, as well as some cardiovascular risk factors, either directly or indirectly through its main target peroxisome proliferator-activated receptor-γ (PPAR-γ), as it will be later clarified. The non-negligible degree of uncertainty about the safety and the exact effects of pioglitazone on the cardiovascular system, both in research and in everyday clinical practice, makes it a very current topic. This is particularly relevant in a worldwide environment where cardiovascular diseases and “diabesity” are the 21st century leading pandemic, with a very high economic impact on the healthcare systems.

To clarify this issue, we reviewed the available literature on the cardiac and vascular effects of pioglitazone, encompassing both *in vitro* and *in vivo* studies in experimental animals and humans, as well as the evidence generated by major randomized clinical trials (RCTs), to condensate and critically evaluate the evidence for an aware clinical use of pioglitazone.

## Pioglitazone and major cardiovascular outcomes: revision of major clinical trials and meta-analyses from the clinician’s point of view

### Atherosclerosis-related events

**Fig. 1:**
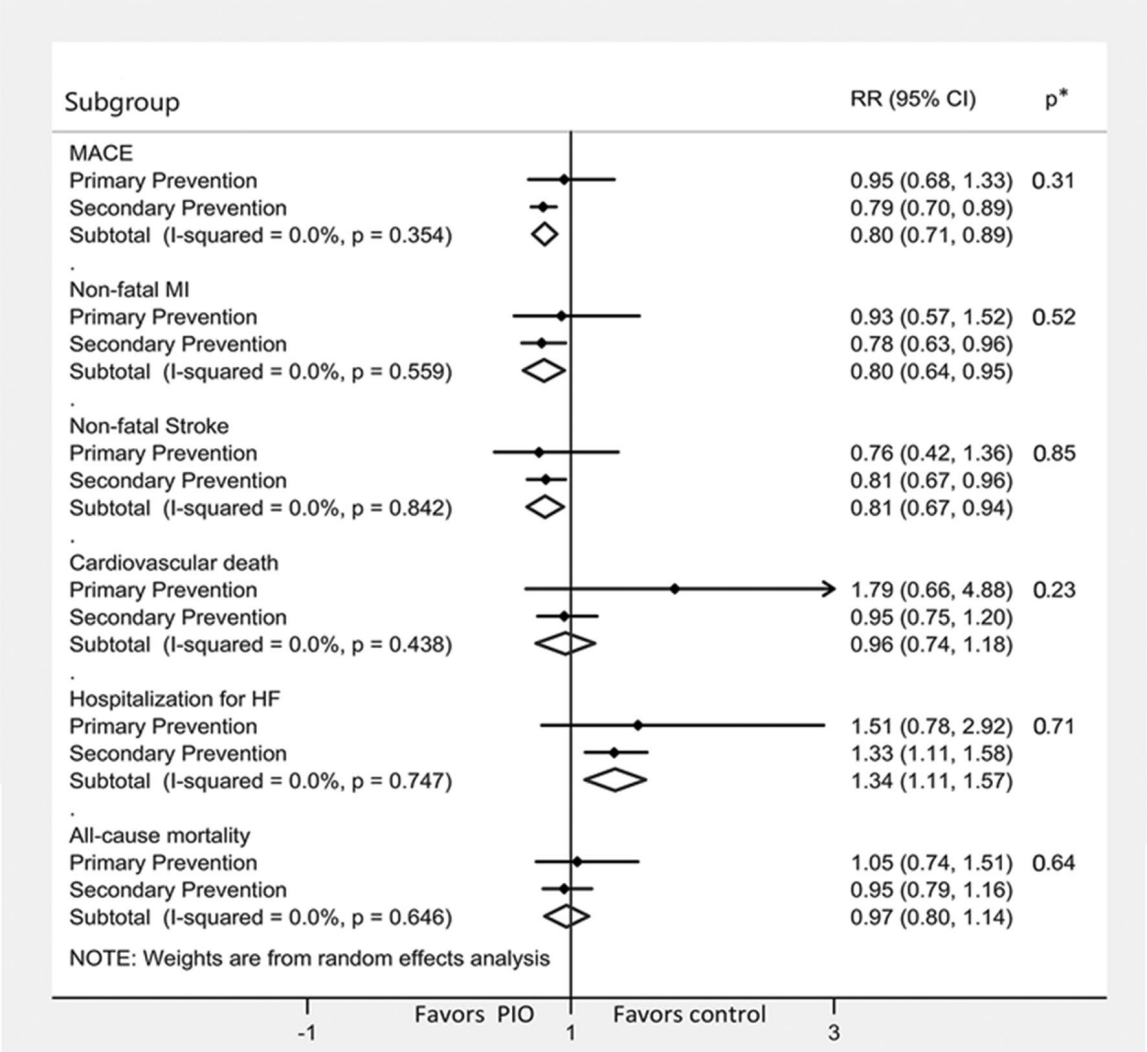
Effects of pioglitazone on cardiovascular endpoints. Metanalysis subgroup analysis on pioglitazone trials on major adverse cardiovascular events (MACE) by primary and secondary prevention. Pioglitazone is associated with a reduction in overall MACE, non-fatal myocardial infarct, and non-fatal stroke, while having a neutral effect on cardiovascular death and all-cause mortality. In overt heart failure, conversely, it is associated with an increase in the risk of hospitalization. Modified with permission from Zhou Y et al. [18]. p* = *p* for heterogeneity between subgroups

Growing evidence suggests a strong protective effect of pioglitazone on atherosclerosis-driven events of either cardiac or cerebrovascular nature. Already in 2005, the PROactive trial enlightened the role of pioglitazone in reducing by 16% the composite risk of all-cause mortality, non-fatal myocardial infarction, and stroke in T2D patients at high risk of macrovascular events (HR 0.84, 95% CI 0.72–0.98) [13]. In the last years, this once underrated result gained increasing attention as several observations pointed out the protective cardiovascular effect of pioglitazone in different clinical settings. In 2017, a meta-analysis of 9 RCTs in individuals with and without CVD (n = 12,026) showed that pioglitazone reduced the risk of major adverse cardiovascular events (MACE, composite of non-fatal myocardial infarction, non-fatal stroke and cardiovascular death) both in pre-diabetic/insulin resistant subjects by 23% (0.77, 0.64–0.93) and in diabetic patients by 17% (0.83, 0.72–0.97) [14]. These results were confirmed by another meta-analysis of 10 RCTs in patients with established cardiovascular disease (CVD) (n = 10,095) wherein pioglitazone reduced recurrent MACE by an impressive 26% (0.94, 0.69–0.92) [15]. Last year, an all-encompassing meta-analysis of 26 RCTs (n = 19,645), including results from the studies TOSCA.IT [6], PPAR [16], and PRIDE [17] and run on a population composed of both diabetics and pre-diabetics and with different CV risk, confirmed a 20% reduction in the risk of MACE (0.80, 0.71–0.89) [18]. A similar risk reduction was seen for two individual MACE components, non-fatal myocardial infarction (20%: 0.80, 0.64–0.95) and non-fatal stroke (19%: 0.81, 0.67–0.94), whereas cardiovascular death (4%: 0.96, 0.74–1.18) and all-cause mortality (3%: 0.97, 0.80–1.14) seemed not affected. In subgroup analyses, the cardiovascular protective effects of pioglitazone were confirmed in patients with pre-diabetes (0.8, 0.6–0.9) and diabetes (0.8, 0.7–1.0). The benefit of pioglitazone was consistently greater in secondary than primary prevention (Fig. 1); however, group differences between patients with and without established CVD were not statistically significant.

Notably, SGLT-2 inhibitors and GLP-1 receptor agonists, which are now acknowledged as the best novel therapies targeting both diabetes and cardiovascular risk, showed a similar magnitude of the effect in reducing MACE by 14% with a HR of 0.86 SGLT-2 inhibitors [19, 20] (though for canagliflozin was not significant [21] and by 16% that is 0.74 to 0.88 for GLP1 receptor agonists [22, 23]. A recent comprehensive umbrella meta-analysis confirmed this point [24]. Although the use of the HR is not free from biases [25], these data show that he effects size of MACE reduction obtained with pioglitazone therapy is comparable to the one observed with the newer (and more expensive—for both the patient and the healthcare system) drugs that recently revolutionized the approach to T2D. With appropriate prescription and titration, this would make of pioglitazone a cost-effective cardioprotective agent in the arsenal of the clinical diabetologist. The aim of the present work is neither to redo a metanalysis nor to systematically review all the studies on cardiovascular outcomes with pioglitazone, which can be found elsewhere; we acknowledge that this is a limit of this manuscript. Our aim is to highlight the effect size of MACE reduction obtained with pioglitazone with a clinical-oriented point of view to help the clinician take aware and critical decisions.

### Heart failure

Large RCTs, including the PROactive [13, 26], RECORD [27], and ADOPT [28] trials, showed an increased hospitalization rate for HF associated with pioglitazone or rosiglitazone treatment. However, in the recent IRIS trial [12] and in a large population-based Asian cohort [29], no difference emerged in the rate of hospitalization for HF among people at low-risk of HF, with proper clinical surveillance and dose titration of pioglitazone treatment. Consistently, a recent meta-analysis confirmed an increased risk of incident HF in patients treated with pioglitazone (1.34, 1.11–1.57), which however appeared limited to those with established CVD (Fig. 1) [18]. These observations suggest that pioglitazone may exacerbate HF particularly in patients with multiple risk factors and/or suffering from subclinical HF. The predominant mechanism, which will be discussed below, may be volume expansion due to renal fluid retention, without alterations in cardiac function or structure [14]. According to this evidence, the current guidelines from the American Diabetes Association (ADA) [30] and the American Heart Association (AHA) [31] recommend that pioglitazone should be used cautiously in patients with symptomatic HF or at risk of acute decompensated HF.

## Effects of pioglitazone on the heart

**Fig. 2:**
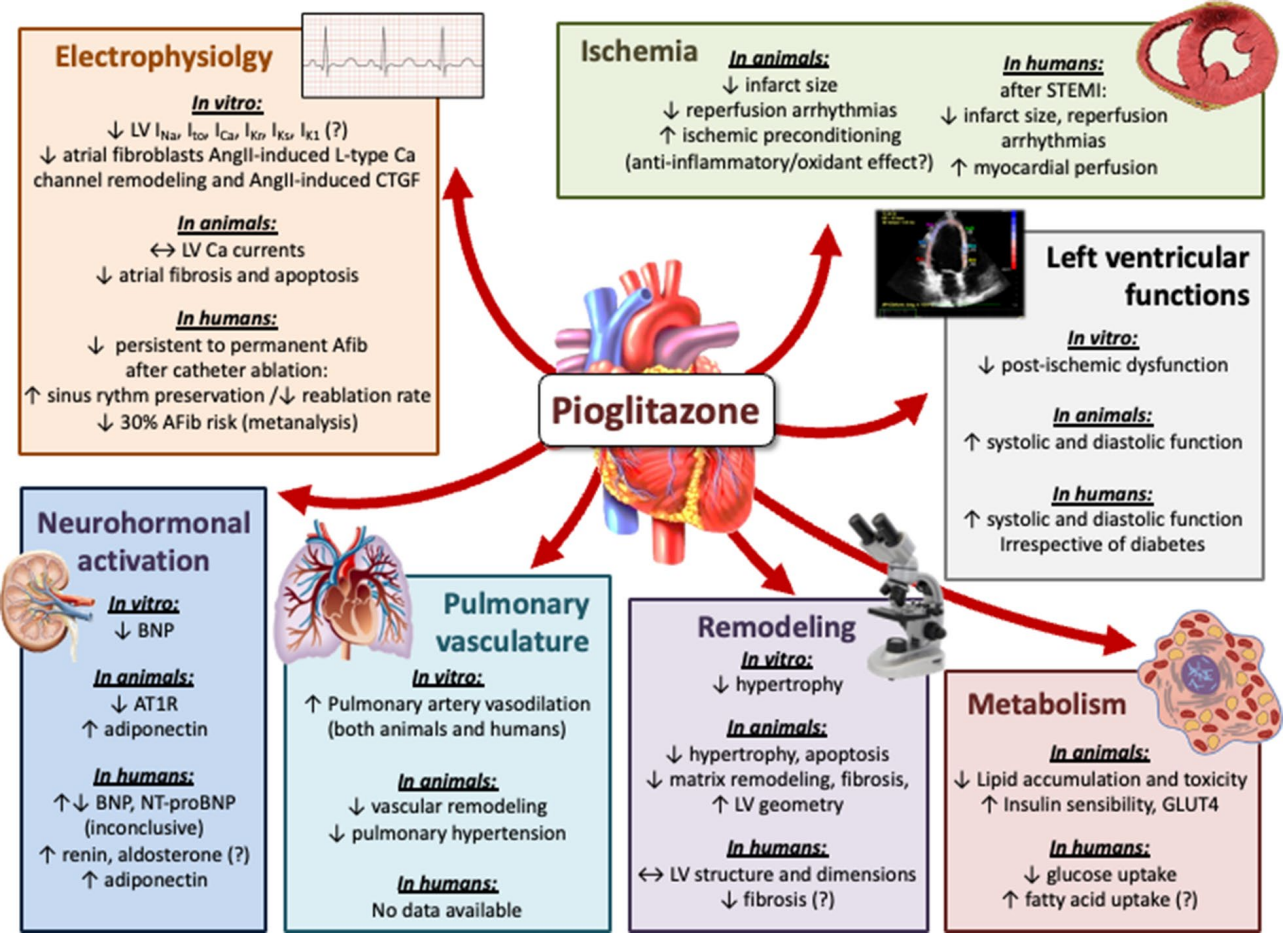
Effects of pioglitazone on the heart. Based on literature review, the effects of the treatment with pioglitazone on diverse cardiac functions are reported. The effects on electrophysiology and arrhythmias, ischemia, left ventricle functions, metabolism, cardiac remodeling, pulmonary vasculature and right ventricle, and on neurohormonal activation are reported. In each section, evidence is subdivided in in vitro, animal, and human studies. For more details, see main text

The effects on cardiovascular outcomes shown by pioglitazone rely on mechanisms that remain largely unknown. The polyhedric effects exerted by this drug can be understood by looking at its main pharmacological target, the nuclear receptor PPAR-γ, a complex transcription factor that is present in almost all tissues and modulates delicate metabolic, inflammatory, and proliferative pathways. Also, PPAR-γ independent mechanisms have been proposed as well, further amplifying the potential cardioactive effects of pioglitazone. The effects of pioglitazone on the heart are schematized in Fig 2.

### Left ventricular systolic and diastolic functions

#### Human studies

A relevant increase in LV ejection fraction (LVEF) and stroke volume was reported in both T2D patients and normal glucose tolerant subjects after 24 weeks of pioglitazone treatment [32], and a borderline increase in LVEF was observed in 30 diabetic patients after 26 weeks of treatment alike [33]. Another study on 24 diabetic subjects randomized to either pioglitazone or placebo confirmed an improvement in stroke volume and LVEF with pioglitazone [34]. With the exception of one study in 88 diabetic patients, which reported no change in diastolic indices with pioglitazone [35], numerous other works on subjects with preserved systolic function reported consistent improvements in diastolic function with amelioration of early-to-atrial mitral flow (E/A) ratio, improvement in tissue Doppler values such as E/e’ and other diastolic parameters, irrespective of the presence of hypertension, diabetes, and/or diastolic dysfunction [17, 34, 36–41].

#### Animal studies

Concordantly with human observations, pioglitazone reduced LV diastolic and systolic dimensions, together with improved contractility in diabetic [42] and non-diabetic murine models [43, 44]. Also, diastolic function was significantly ameliorated as several diastolic parameters were improved by the treatment in diabetic models [45]. Notably, pretreatment of mice with pioglitazone improved both systolic and diastolic functions regardless of the etiologic cause, being either ischemia [44, 46], pressure overload or high-fat diet [43]. It also reduced aortic valve calcification in non-diabetic, hypercholesterolemic rats [47].

### Cardiac remodeling

#### Human studies

Few studies reported the effects of pioglitazone on changes in cardiac size and function, the results being somewhat controversial. Mean aortic diameter and left atrial systolic and diastolic volumes significantly decreased after 6 months of therapy with pioglitazone in 49 T2D subjects [38]. However, different authors reported that pioglitazone treatment was associated with a 17% increase in left atrial volume in 30 T2D patients after 26 weeks of pioglitazone treatment [33], while others [36] found no change in absolute values of left atrium volume and LV end-diastolic diameter in 30 non-diabetic patients with essential hypertension after 6 month pioglitazone treatment. The different response may be due to the rather small sample size of these observation, coupled with the different baseline characteristics of the patients. As such, there is still uncertainty about pioglitazone effect on cardiac remodeling in humans, and mechanisms are still unknown. Noteworthy, pioglitazone reduces plasma collagen III in humans [37], possibly exerting anti-fibrotic effects.

#### Animal studies.

*In vitro* and *in vivo* observations showed pioglitazone to have anti-hypertrophic potential in cultured mice cardiomyocytes via inhibition of vascular endothelial growth factor (VEGF) [48] and inhibition of pressure overload-induced increases in cardiac wall thickness and myocyte diameter in wild-type mice. Moreover, it reduced the increase in the heart weight-to-body weight ratio in heterozygous non-diabetic PPAR-γ-deficient mice [49], suggesting PPAR-γ independent activity. Moreover, it was shown to have beneficial long-term effects on cardiac remodeling in non-diabetic stroke-prone rodents by normalizing echo-assessed left ventricle (LV) geometry, reversing concentric remodeling, and decreasing myocyte diameter, interstitial fibrosis and number of myofibroblasts [50]. These effects are coupled with the inactivation of well-established transcription factors involved in cardiomyocyte hypertrophy NFATc2 and NF-kB/p65 [51] that coordinate a program of reactivation of fetal ventricular gene expression profiles typical of (adverse) cardiac remodeling [52]. In this scenario, convincing data support the anti-fibrotic effect of pioglitazone in cardiac remodeling in several studies documenting a consistent reduction of histologically proven cardiac fibrosis after pioglitazone treatment [53, 54]. Particularly, in a model of non-diabetic ischemia-reperfusion injury, pioglitazone reduced the synthesis of extracellular matrix, particularly collagen I and III, tissue inhibitor of metalloproteinase (TIMP)-1, and matrix metalloproteinase (MMP)-2 in cardiac fibroblasts, a pathway involving the PPAR-γ-dependent inhibition of NF-*k*B [55]. Also, inhibition of connective tissue growth factor (CTGF) expression [50] and attenuated angiotensin II-induced cardiac fibrosis by reduced myocardial macrophage infiltration were reported [56]. Other recent works observed reduced fibrosis through reduced expression of TGF-β in macrophages from diabetic mice [57] through PTEN [58], Smad3 activation [59], and the newly discovered SIRT3/β-catenin/PPAR-γ axis, which prevent cardiac fibroblasts from transdifferentiating into myoblasts [60]. Based upon these observations, PPAR-γ agonists have been tested as potential therapeutic agents in the suppression of collagen synthesis in the lung and the liver, both *in vitro* and *in vivo* [61], with encouraging results.

### Electrophysiology and arrhythmias

#### Human studies

Several trials demonstrated that pioglitazone treatment might have beneficial influence in atrial fibrillation (AFib). Although it did not influence the recurrence of persistent AFib after successful electrical cardioversion in T2D subjects [62], it proved effective in reducing the rate of progression from persistent to permanent AFib [63], improved the preservation of sinus rhythm and reduced the reablation rate in diabetic patients with paroxysmal AFib after catheter ablation [64]. These results were confirmed by one metanalysis conducted in 2017 on more than 1,30,000 diabetic patients in which pioglitazone treatment was associated with a 30% risk reduction of developing AFib (OR = 0.73, p = 0.0003) with reduced risk for both new-onset AFib (OR = 0.77, p = 0.002) and AFib recurrence (OR = 0.41, p = 0.002) [65]. As a consequence, pioglitazone has been proposed for AFib primary and secondary prevention in diabetes [66]. The mechanism might rely either on its anti-fibrotic effects (as discussed above) or on the reduction of glycation end products [63], although this is not clear.

#### Animal studies

Pioglitazone prevented AFib by increased membrane potential through electrophysiological remodeling both reduced angiotensin 2-induced potassium channel remodeling on isolated non-diabetic animal atrial myocytes and attenuation of angiotensin 2-induced L-type calcium channel remodeling [67]. Furthermore, it can reduce atrial fibrosis and normalize interatrial conduction time [68] by suppression of angiotensin 2-induced CTGF expression and proliferation in atrial fibroblasts from both diabetic and non-diabetic animals, with mechanisms encompassing various signaling pathways such as TGF-β1/Smad2/3 and TGF-β1/TRAF6/TAK1 [69, 70], p-ERK1/2, p-JNK, the mitochondrial apoptotic signaling pathway, and the caspase system [67]. The mechanism is independent from the metabolic effects of the drug, and seemingly acts through the PPAR-γ receptor [67], but this was never directly demonstrated. In canine ventricular myocytes [71], high doses of pioglitazone altered a wide variety of ion currents in a concentration-dependent manner, namely I_Na_, I_to_, I_Ca_, I_Kr_, I_Ks_, I_K1_, and the ATP-sensitive potassium current. However, it is not likely that normally dosed pioglitazone can significantly alter ventricular electrogenesis in healthy humans, apart from overdose or inherited or acquired long QT syndrome, wherein it might favor arrhythmias and particularly early afterdepolarizations. However, no sudden cardiac death has ever been reported with pioglitazone therapy.

### Energetic metabolism

#### Human studies

In cardiomyocytes, just like in adipose tissue, the genes activated by PPAR-γ stimulate lipid uptake and adipogenesis [72]. As such, alterations in its cardiac expression cause disturbances in glucose and fatty acids metabolism, contributing to intracellular triglyceride accumulation and cardiac lipotoxicity [73] with resulting significant LV dysfunction [74]. In humans, pioglitazone treatment enhances insulin-stimulated myocardial glucose uptake as measured through ^18^FDG PET across the whole spectrum of glucose tolerance [32, 41, 75], including T2D with coronary artery disease [76]. This is related to a reduction of serum free fatty acids [77, 78], since pioglitazone promotes fatty acid transport into the cardiomyocytes inducing the expression of fatty acid-binding protein 4 (FABP4) and fatty acid translocase (FAT)/CD36 in capillary endothelial cells [79]. However, the clinical implications are poorly known, and more studies are required. Interestingly, since right ventricular dysplasia has been linked to changes in PPAR-γ-dependent pathways in cardiomyocytes energetic metabolism leading to myosin dysfunction [80], pioglitazone has been proposed as a possible therapy for this condition, by acting through alterations in PPAR-γ-dependent Wnt/*β*-catenin canonical pathway [81].

#### Animal studies

Due to low myocardial PPAR-γ expression, in vivo effects of TDZs on cardiac metabolism are generally thought to be indirect and secondary to their lipid lowering properties [82], and a direct regulation of cardiac metabolism by PPARγ remains a subject of debate. Pioglitazone can induce lipid accumulation in the heart of rats despite concurrent reduction in plasma free fatty acids concentration, thus suggesting a direct action of PPARγ agonist on the cardiomyocyte [83] and seemingly a de novo synthesis of ceramides [84]. More recently it has shown that tissue-specific loss of PPARγ alters heart function and induces myocardial hypertrophy [85, 86] with mitochondrial oxidative damage [86] although no effect on gene expression controlling lipid and glucose metabolism at baseline was observed [85]. This modulation of cardiomyocyte metabolism might have a direct effect on the functions of the heart. Indeed, PPAR-γ transgenic mice over-expressing PPAR-γ develop a dilated cardiomyopathy with evidence of increased lipid and glycogen stores, increased mRNA levels of genes for fatty acids oxidation, and distorted architecture of the mitochondrial inner matrix [87]. In contrast, treatment with PPAR-γ agonists improved heart function in rodent models of lipotoxic dilated cardiomyopathy with unclear mechanisms [88, 89], since rosiglitazone treatment of wild-type mice reduced expression of PPARγ targets [87].

### Ischemia–reperfusion injury

#### Human studies

Several observations reported a protective effect on myocardial ischemia-reperfusion injury. In diabetic patients with ST-elevation MI (STEMI), pretreatment with pioglitazone resulted in better myocardial reperfusion. This was described by blush score, slow flow/no-reflow phenomenon, resolution of ST elevation, and lesser reperfusion injury as defined by absence of reperfusion arrhythmias, better improvement of LVEF, and lower peak creatin kinase levels [90].

#### Animal studies

In animals, available evidence showed reduction in infarct size in mice and rats acutely pretreated with pioglitazone [91–95] through enhanced anti-oxidant superoxide dismutase and glutathione peroxidase concentrations [93], activation of ERK and COX2 [96], and reduced cardiomyocyte apoptosis via enhanced Bcl-2 protein expression, reduced Bax and caspase 3 protein expression [46]. Also, pioglitazone attenuated reperfusion arrhythmias after ischemic-reperfusion injury in diabetic rats [97], and ameliorated the deteriorated ischemic preconditioning found in diabetic animals [98, 99]. The treatment with PPAR-γ agonists decreased the expression of pro-inflammatory markers and reduced accumulation of neutrophils and macrophages in animal reperfused myocardium possibly through activation of NF-kB [100], an observation confirmed by a recent study in PPAR-γ knock-out mice [55]. As such, supported by the observation that an anti-inflammatory effect is coupled with protection from ischemia in other tissues alike -namely liver [101], kidney [102], and gut [103]-, we can hypothesize that pioglitazone might in part protect from ischemia–reperfusion injury via an anti-inflammatory effect. Still, the drug might also exert its protective effect on ischemia via a metabolic action mediated by cardiomyocyte-derived adiponectin [104], which has been observed to be protective during ischemia [105].

### Neurohormonal activation

#### Human studies

Pioglitazone can modulate diverse neurohormonal systems that are known to positively affect the cardiovascular system. In obese subjects with metabolic syndrome, the sympathetic nervous system response to an oral carbohydrate load is enhanced by pioglitazone, with significant increase in overall norepinephrine spillover response [106], despite no changes in resting sympathetic drive or norepinephrine disposition [39]. It can also increase adiponectin release [104] with beneficial effects on diastolic function in hypertensive patients [36]; notably, this is one of the mechanisms proposed for the beneficial effects of SGLT-2 inhibitors. One trial on 94 diabetic patients undergoing coronary angioplasty reported a significant reduction of brain natriuretic peptide (BNP) levels with pioglitazone with respect to other hypoglycemic treatments [17]. Similarly, lower BNP values [107] in 223 diabetic subjects reported with the combined sulphonylurea-pioglitazone therapy with respect to other medications. On the contrary, two smaller studies observed a significant increase in BNP values during pioglitazone treatment [108, 109], with elevated BNP values at baseline predicting a subsequent increase [109], suggesting that pioglitazone negative effect on BNP may only occur when cardiac function is already altered. One study on 30 diabetic subjects revealed an increase in N-terminal-proBNP (NT-proBNP) after 6 months of treatment with pioglitazone with a parallel slight increase in LV and left atrial volumes [33], leaving the question open.

#### Animal studies

At the cellular level, it can down-regulate the expression of angiotensin receptor type 1 (AT1-R) in neonatal non-diabetic rat cardiac fibroblasts, thus reducing the angiotensin-induced cardiac fibrosis and remodeling [110]. This might explain the slight increase of renin activity observed with pioglitazone in healthy subjects [111]. Also, PPAR-γ activators directly inhibited strain- and reperfusion-induced BNP expression in non-diabetic cultured cardiomyocytes and cardiac fibroblasts [55, 112].

### Pulmonary vasculature and right ventricular function

#### Human studies

In human pulmonary vascular wall, PPAR-γ receptors are normally expressed by endothelial and smooth muscle cells [113], while their expression appears diminished in patients with primary and secondary pulmonary hypertension [114]. In isolated pulmonary arteries from healthy subjects, pioglitazone induced a concentration- and time-dependent relaxation with a mechanism involving nitric oxide (NO) production that is only partially dependent on vascular endothelium and dependent on PPAR-γ activity [115].

#### Animal studies

Similarly to humans, several animal studies reported improved pulmonary hypertension and right ventricle (RV) hypertrophy [116], pulmonary hypertension prevention [117, 118] and pulmonary vasculature remodeling [119] by PPAR-γ-dependent inhibition of transcription factors NFAT and NF-*k*B [120]. The drug might also have a role in reducing right ventricular dysfunction [121, 122], via inhibition of NF-*k*B and NFAT in cardiomyocytes of the failing RV [121] and normalize epigenetic and transcriptional regulation factors [123] primarily related to insulin resistance, disturbed lipid metabolism and mitochondrial morphology/function [121, 124, 125].

## Effects of pioglitazone on the vascular system

**Fig. 3:**
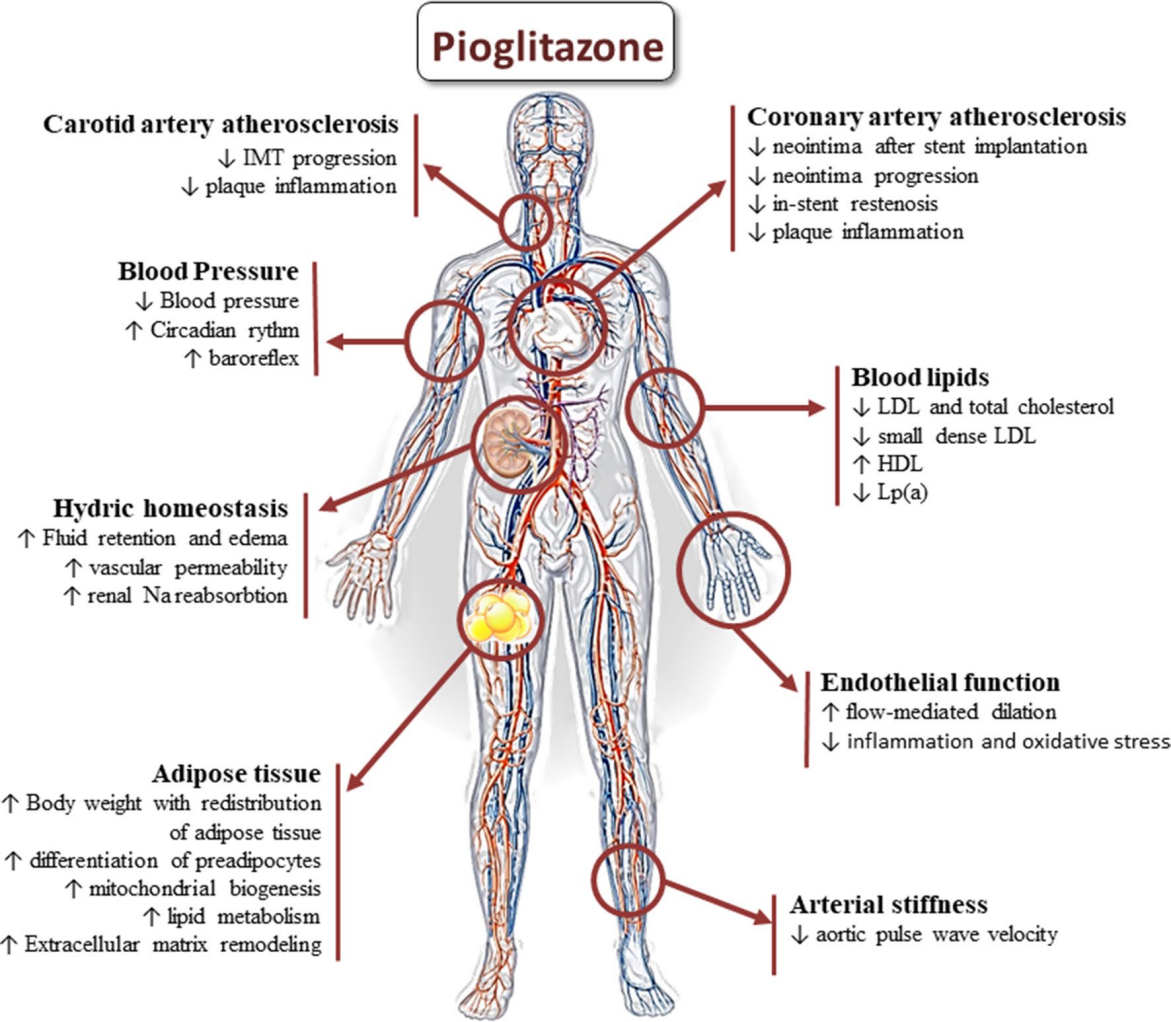
Effects of pioglitazone on the vascular system and on cardiovascular risk factors. The effects of the treatment with pioglitazone on the vasculature and on modifiable risk factors are illustrated. The effects on atherosclerosis, endothelial function and blood pressure are reported, together with hydro-electrolyte homeostasis, the effects on the adipose tissue, and on blood lipids. For more details, see main text

Several human and animal studies suggest a beneficial role for pioglitazone on the vascular system, particularly on atherosclerosis reduction. The effects of pioglitazone on the vascular system are schematized in Fig. 3.

### Human studies

Pioglitazone can reduce aortic pulse wave velocity (PWV) irrespective of the presence of diabetes [126], and slow progression of carotid intima-media thickness in T2D subjects as measured with either ultrasonography [127–129] or fluorodeoxyglucose positron emission tomography/computed tomography (FDG-PET/CT) [130]. This was also observed both in non-diabetic patients with stable coronary artery disease and carotid plaques [130] and in glucose-intolerant patients [131]. Furthermore, pioglitazone positively affects plaque remodeling by inducing reduced coronary neointima hyperplasia in diabetic subjects [132], diminished intravascular ultrasonography (IVUS)-assessed neointima volume after stent implantation in non-diabetic subjects [133, 134], and slowed progression of coronary atherosclerosis as quantified through IVUS in comparison to glimepiride in 360 diabetic subjects with coronary atherosclerosis [135]. Noteworthy, it significantly decreased in-stent restenosis after coronary stent implantation in T2D patients [136] as also confirmed by a later metanalysis [137]. On the contrary, no beneficial effect was seen by Lee et al. [138] neither on in-stent restenosis nor on coronary plaque volume measured by IVUS, though this might be due to the low administered dose (15 mg/die). Two metanalysis studied the effect of pioglitazone in reducing the in-stent restenosis and coronary atherosclerosis. The first by Zhang MD et al. [139] in 2014 reported a neutral effect, while the second by Zhao SJ et al. [140] in 2016 reported a beneficial effect for pioglitazone both on in-stent restenosis (0.48, 0.35–0.68) and target lesion revascularization (0.58, 0.38–0.87). Furthermore, several studies investigated the effect of pioglitazone on endothelial function in humans through flow-mediated dilation in brachial artery in either diabetic [141, 142], glucose intolerant [143, 144], or non-diabetic [126] patients demonstrating a significant beneficial effect for pioglitazone on ameliorating endothelial dysfunction irrespective of glucose tolerance phenotype, a result further confirmed by a recent metanalysis [145]. Intriguingly, this appears to be independent of its insulin-sensitizing action [146, 147], and possibly related to the known increase in adiponectin levels [143]. Nonetheless, in the study by Davidson et al. [148] the reduction of carotid intima-media progression was related to an increase in HDL cholesterol at 24 weeks of treatment, suggesting that even other factors might be at play.

The mechanisms seemingly rely on anti-inflammatory and anti-oxidative effects, reducing carotid plaque inflammation in patients with higher baseline inflammation [130], reducing circulating natural killer cells, diminishing IL-6 and monocyte chemoattractant protein (MCP)-1 -also known as GDF15- levels, downregulating chemokine receptor 2 at 48 h after stent implantation in T2D [134]. With pioglitazone treatment, lower IL-10 levels at ten days after implantation was achieved, together with possible anti-proliferative effects on vascular smooth muscle cells [134]. This hypothesis is supported by the correlation of reduced aortic PWV with the reduction of clinical and biohumoral indices of inflammation in non-diabetic subjects with rheumatoid arthritis [126].

### Animal studies

In animals, PPAR-γ activation has been implied in inhibiting VEGF-induced angiogenesis [149] and in decreasing the inflammatory response of several cardiovascular structures, particularly endothelial cells [150]. Pioglitazone can also protect endothelial progenitor cells from hyperhomocysteinemia via reduced reactive oxygen species (ROS) production by NADPH and PKC downregulation [151], inhibition of TGFβ1-induced mitochondrial activation and vascular smooth muscle cell proliferation by regulating two glucose metabolism-related enzymes, platelet isoform of phosphofructokinase (PFKP, a PPAR-γ target, via miR-331-5p) and protein phosphatase 1 regulatory subunit 3G (PPP1R3G, a Smad3 target) [152]. Further, pioglitazone treatment suppressed excess lipid accumulation and superoxide production in the aorta in an angiotensin II-induced rat model of hypertension and retarded the progression of atherosclerosis [153]. Moreover, PPAR-γ agonists have been shown to down-regulate both basal and TNF-α-induced Receptor for the advanced glycation end products (RAGE) expression in endothelial and mesangial cells [154], and to reduce the production of endothelin-1 and ROS by directly blocking cyclo-oxygenase (COX)-2 [155], exerting a protective effect on endothelial dysfunction through anti-oxidant activity. Notably, in spontaneously hypertensive rats, pioglitazone improved NO availability through increased NO synthetase (NOS) expression and AT2R with an overall significant blood pressure reduction effect [156].

## Effects of pioglitazone on cardiovascular risk factors

### Body weight, adipose tissue, and blood lipid profile

Pioglitazone treatment is associated with an increase in insulin sensitivity in diabetic subjects, notwithstanding its neutral [157] or increasing effect in body weight of about 2.5–3 kg [158]. This is due to a change in adipose tissue distribution with reduced visceral fat in favour of subcutaneous fat [157, 159–161]; in non-diabetic individuals it promotes an increase in total body fat content with preferential accumulation in the lower body parts and reduction in waist-to-hip ratio [162]. Both in animals [163] and humans [161, 162, 164], pioglitazone increases the number and the activity of small adipocytes promoting differentiation of preadipocytes to adipocytes in visceral and subcutaneous adipose tissue stimulating glucose uptake, storage, and metabolism [165, 166]. In adipose tissue, pioglitazone induces mitochondrial biogenesis, the synthesis of mitochondrial lipid metabolism enzymes [167, 168], and alters extracellular matrix and cytoskeletal proteins [167]. A deeper characterization of the metabolic effects of pioglitazone in human adipose tissue is needed, since some cardioprotective mechanisms might involve adipocytokines. Furthermore, pioglitazone treatment determines a reduction in total cholesterol, LDL-cholesterol, triglycerides, and plasma free fatty acids; it also converts small dense atherogenic LDL particles into larger ones, and increases HDL-cholesterol [169–171]. Remarkably, pioglitazone treatment reduces Lp (a) [169] as well as procoagulant factors [172]. According to the current knowledge, all these mechanisms are expected to provide cardiovascular benefits.

### Blood pressure

#### Human studies

A slight but consistent blood pressure lowering effect has been consistently reported with pioglitazone [39, 40, 128, 141, 173–175], achieving an average reduction by 3–5 mmHg in systolic blood pressure after 12 months of treatment. Interestingly, pioglitazone at the oral dose of 30 mg/die was effective in normalizing both blood pressure and serum potassium levels in one case of resistant hypertension due to primary hyperaldosteronism in a diabetic subject [176]. In fact, a slight antagonistic effect on the angiotensin receptors has been observed, with a consequent increase in plasma renin activity [111]. Aside from its direct effect on absolute blood pressure values, its protective effect is seemingly dependent upon a reassessment of the cardiovascular circadian clock, being PPAR-γ a main component of the vascular system circadian regulation, known to be altered by metabolic pathologic conditions such as obesity and diabetes [177, 178]. In diabetic patients, pioglitazone is known to re-establish the circadian rhythm of blood pressure from a “non-dipper” to a “dipper” pattern, restoring the nocturnal decline in blood pressure in parallel with reduction in the HOMA index [179, 180]. Decreased blood pressure and enhanced baroreflex sensitivity with pioglitazone was reported after an oral carbohydrate load in obese subjects with metabolic syndrome [106], and there is initial evidence that this drug might have a beneficial role in restoring the sympatho-vagal balance in diabetic autonomic cardiomyopathy [181].

#### Animal studies

The observations on humans are confirmed in animal studies [182] in hypertensive models [156, 183] wherein a reduction in hypertension-related end organ damage, including (LV) hypertrophy [184], proteinuria [185], and white matter lesions and cognitive impairment were reported [186]. One plausible determinant of the anti-hypertensive effect of piogliazone is the attenuation of vascular contractility via a NO-independent mechanism that directly involves intracellular calcium handling through the inhibition of L-type Ca^2+^ currents in vascular smooth muscle cells [187] independently and synergistically with insulin [188]. Pioglitazone can directly regulate Rev-erb-α, a transcription factor influencing Brain and Muscle Arnt-Like protein (BMAL)-1 [178, 189], a key clock-gene involved in cardiovascular rhythmicity [190] and possibly accounting for its effect on the circadian clock. Yet, whether the main target cells of pioglitazone are vascular smooth muscle cells, liver, peripheral adipose tissue, or perivascular adipose tissue is still matter of debate [178].

### Fluid and electrolyte homeostasis

Oedema was early recognized as a frequent and potentially clinically relevant side effect of pioglitazone, possibly precipitating HF.

#### Human studies

Fluid retention and peripheral oedema were reported in 4–7% of pioglitazone-treated patients, that is 3–4 times more frequent than placebo, an effect even greater in combination with other oral anti-hyperglycemic treatments (up to 15–18%) -and especially insulin therapy (~22%) [191, 192]. The mechanism, which can be not always counteracted by diuretic therapy and that is relieved by drug withdrawal, is seemingly multifactorial and still poorly understood. In fact, pioglitazone can inhibit L-type calcium currents [187] thus possibly having an effect similar to that of dihydropyridine calcium channel blockers. It also increases VEGF activity [193] which leads to capillary wall permeabilization. Yet, renal sodium-water increased reabsorption plays a major role, accounting for up to 80–90% of thiazolidinediones-related fluid retention [194]. In human proximal tubule cells this is mainly achieved through enhanced expression of sodium-hydrogen exchangers (NHE3) [195–198], proximal tubule aquaporine (AQP) 1 and 7 channels [196], and possibly a direct pioglitazone-driven sodium reabsorption [111]. Moreover, insulin has been known to stimulate sodium absorption along various nephron segments, potentially acting synergistically with pioglitazone [199] and possibly accounting for the greater incidence of oedema formation during combined therapy.

#### Animal studies

Animal studies report conflicting results on a reduction in glomerular filtration rate with an effect size that was largely overcome by tubular reabsorption [200, 201]. This result was later confirmed by the potent PPAR*γ* agonist, farglitazar (GI262570), which induced plasma volume expansion in normal rats with significant reduction of hematocrit, hemoglobin, and serum albumin concentration but no measurable effect on GFR, renal blood flow, or filtration fraction [202]. These results suggest that thiazolidinediones-induced fluid retention occurs mainly by tubular rather than glomerular mechanism. Several works suggest that it might induce plasma volume expansion acting either on the cortical collecting duct via enhanced expression of the γ subunit of epithelial Na channel (ENaC) [203], or on the renal proximal tubule through sodium-coupled bicarbonate transporters, sodium-hydrogen exchangers, NHE3 [200], and basolateral rheogenic Na^+^/HCO3 cotransport (NBCe1) [204]. Finally, also the Henle’s loop bumetanide-sensitive Na–K–2Cl cotransporter (NKCC) may be upregulated by pioglitazone [205]. However, the exact mechanisms accounting for the renal effects of pioglitazone remain unclear.

## Clinical considerations

Pioglitazone has some adverse effects that warrant caution in at risk patients and limited its use in clinical practice, including body weight gain, peripheral oedema, increased congestive HF risk, decreased bone mineral density, dilution anemia, and possibly increased risk for bladder cancer [2]. Despite these safety concerns, pioglitazone has recently gained renewed popularity after several clinical trials reporting reduction in atherosclerosis, AFib, and atherosclerosis-related events. Of note, it positively modulates numerous cardiovascular functions and risk factors such as systemic blood pressure, blood lipids, and adipose tissue physiology, thus leading to slowed atherosclerosis progression. It can also restore cardiovascular rhythmicity, increase LV systo–diastolic functions, protect against myocardial ischemia and fibrosis, as well as possibly reduce pulmonary hypertension. The exact mechanisms remain unclear, seemingly encompassing anti-inflammatory, anti-oxidative, anti-hypertrophic, anti-fibrotic, and vasodilatory effects -both PPAR-γ dependent and independent, which to date remain rather unclear in their precise molecular mechanisms. In this scenario, it is important to underscore that the treatment with pioglitazone is low-cost, widely available, without significant drug interactions, and with little side effects such as mild body weight gain and risk of oedema formation. Even without a direct negative influence on cardiac function, the retentive effect of pioglitazone may be clinically relevant, as it can possibly lead to overt HF by unmasking previously undiagnosed cardiac dysfunction. However, with aware appropriate prescription and titration in those with or at high risk for acute decompensated HF, the above described polyhedric cardiovascular benefits of pioglitazone identify this drug as a useful tool in the arsenal of the clinical diabetologist, particularly when used in combination with SGLT-2 inhibitors. In fact, the depletion of extracellular fluid volume by SGLT-2 inhibitors is expected to contrast the expansion of fluid volume by TZDs, while the beneficial effects on cardiovascular prevention of the two drug classes are potentially additive or even multiplicative, due to their different mechanisms of action. For these arguments, TZDs and SGLT-2 inhibitora have been proposed by DeFronzo et al. [206, 207] as ideal partners in combination therapy. Therefore, a diabetic subject with insulin resistance, non-controlled or non-dipper hypertension, and at high risk for or with multi-vessel atherosclerosis perfectly fits the ideal candidate for pioglitazone. The concomitants reduction of paroxysmal AFib incidence of recurrence further support the therapeutic choice.

## Concluding remarks

Pioglitazone is a PPAR-γ agonist acting as an insulin sensitizer with cardiovascular protective potential, despite the risk of fluid imbalance in overt HF. The pleiotropic and beneficial effects on cardiovascular risk factors are exerted above and beyond glycemic improvement, encompassing myocardial beneficial effects both directly, through anti-fibrotic and anti-remodeling actions, and indirectly, by improving vascular homeostasis. Accordingly, clinical trials have observed its capacity of slowing atherosclerosis progression, possibly accounting for the reduction in cardio- and cerebrovascular events. The mechanisms involved might rely either on cardiac and vascular anti-remodeling properties (endothelium protective, inflammation-modulating, anti-proliferative and anti-fibrotic properties) and/or on metabolic (adipose tissue metabolism, increased HDL cholesterol) and neurohormonal (renin-angiotensin-aldosterone system, sympathetic nervous system, and adiponectin) modulation of the cardiovascular system. These polyhedric beneficial effects make pioglitazone a useful tool in the arsenal of the clinical diabetologist, especially in the insulin-resistant, hypertensive patient at high cardiovascular risk.

## Data Availability

Not applicable.

## Abbreviations

AFib: Atrial fibrillation
AT-R1: Angiotensin receptor type 1
BNP: Brain natriuretic peptide
COX: Cyclo-oxygenase
CVD: Cardiovascular disease
E/A ratio: Early-to-atrial mitral inflow Doppler wave ratio
ENaC: Epithelial sodium channel
GLP-1: Glucagone like peptide 1
HDL: High density lipoproteins
HF: Heart failure
IVUS: Intravascular ultrasonography
LDL: Low density lipoproteins
Lp (a): Lipoprotein a
LV: Left ventricle
LVEF: Left ventricular ejection fraction
MACE: Major adverse cardiovascular events
NF-kB: Nuclear factor k-B
NO: Nitric oxide
NOS: Nitric oxide synthetase
NT-proBNP: N terminal-pro brain natriuretic peptide
PPAR-γ: Peroxisome proliferator-activated receptor gamma
PWV: Pulse wave velocity
RAGE: Receptor for the advanced glycation end products
RCTs: Randomized clinical trials
ROS: Reactive oxygen species
RV: Right ventricle
SGLT-2: Sodium–glucose cotransporter 2
STEMI: ST elevation myocardial infarction
T2D: Type 2 diabetes
TDZs: Thiazolidinediones
VEGF: Vascular endothelial growth factor
